# The role of VDR and BIM in potentiation of cytarabine–induced cell death in human AML blasts

**DOI:** 10.18632/oncotarget.8998

**Published:** 2016-04-26

**Authors:** Jonathan S. Harrison, Xuening Wang, George P. Studzinski

**Affiliations:** ^1^ Department of Medicine, University of Missouri, Columbia, Missouri 65212, USA; ^2^ Department of Pathology & Laboratory Medicine, Rutgers New Jersey Medical School, Newark, New Jersey 07103, USA

**Keywords:** acute myeloid leukemia, cytarabine, vitamin D analog, plant antioxidant, Bim

## Abstract

Acute Myeloid Leukemia (AML) has grave prognosis due to aggressive nature of the disease, the toxicity of standard treatment, and overall low cure rates. We recently showed that AML cells in established culture treated with cytarabine (AraC) and a differentiation agent combination show enhancement of AraC cytotoxicity. Here we elucidate molecular changes which underlie this observation with focus on AML blasts in primary culture. The cells were treated with AraC at concentrations achievable in clinical settings, and followed by the addition of Doxercalciferol, a vitamin D2 derivative (D2), together with Carnosic acid (CA), a plant-derived antioxidant. Importantly, although AraC is also toxic to normal bone marrow cell population, the enhanced cell kill by D2/CA was limited to malignant blasts. This enhancement of cell death was associated with activation of the monocytic differentiation program as shown by molecular markers, and the increased expression of vitamin D receptor (VDR). Apoptosis elicited by this treatment is caspase-dependent, and the optimal blast killing required the increased expression of the apoptosis regulator Bim. These data suggest that testing of this regimen in the clinic is warranted.

## INTRODUCTION

Acute Myeloid Leukemia (AML) if untreated is a rapidly lethal disease, yet limited progress has been achieved in the past three decades for improving the long-term disease-free survival of AML patients using standard chemotherapy regimens [1]. Therefore the development of new effective therapeutic regimens represents a high priority. DNA damaging agents such as cytarabine (AraC) often produce brief remissions of AML; conversely, very toxic therapy and allogeneic hematopoietic transplantation, which can be curative, can only be offered to a subset of the most robust patients. Thus, modifications of standard chemotherapy are being developed, but none have so far supplanted the decades-old existing regimens.

AraC has been combined with other agents, most commonly anthracyclines such as daunorubicin, idarubicin and epirubicin; the anthracenedione mitoxantrone, and the epipodophyllotoxin etoposide (eg, [2, 3]). However, these and other AraC combinations are also extremely toxic, thus adding to the overall systemic toxicity of the therapy, for instance the cardiotoxicity of the anthracyclines, or of azacytidine combined with AraC [4].

Vitamin D derivatives (VDDs) such as the physiologically active form, the 1,25-dihydroxyvitamin D3 (1,25D) or synthetic analogs, have long been considered to have anti-neoplastic properties, based on epidemiological studies [5–8] and tissue culture evidence (eg, [9–12]). The initial observations of anti-cancer activity of VDDs were made over 30 years ago, in which proliferation of calcitriol-treated cultured melanoma cells was shown to be inhibited [9]. Shortly thereafter, Suda laboratory reported that murine and human AML cells in established culture undergo differentiation [10, 11]. The anti-leukemic effect is manifested by acquisition of the monocyte/macrophage–like phenotype, and the associated cell cycle arrest of the cells exposed to VDDs [13, 14]. The concentration of VDDs required to achieve this in cell culture far exceeded the concentrations of circulating 1,25D, but provided a “proof of concept”. However, this has been thus far incapable of practical application, since in vivo such concentrations of VDDs would cause hypercalcemia incompatible with life. Similarly, combinations of VDDs with other compounds have so far not met with a success in the clinic [15–17].

This laboratory has previously demonstrated that when Carnosic acid (CA), a plant–derived polyphenol with anti-oxidant activity [18], is added to 1,25D or other VDDs, it markedly increases their pro-differentiation activity in HL60 cells, a human AML cell line in established culture [19]. Recently, we found that a combination of CA with Doxercalciferol (D2), a low calcemic vitamin D2 derivative already approved for human administration in dialysis patients, can increase the toxicity of AraC to AML cells in established culture (AraC-D2/CA treatment). However, multiple passages of human cells in culture are known to introduce phenotypic and/or genetic changes and thus cell lines may not be representative of cells in vivo. Therefore, we now undertook a translational study with freshly obtained blasts or normal bone marrow cells placed in primary culture to investigate the molecular changes underlying this potentially therapeutic effect. AML cells in established culture were also used to supplement the scarce material of ex vivo samples.

Here we provide evidence that when treatment of ex vivo blasts with AraC is followed by D2/CA combination there is significantly increased death of the malignant cells dependent on vitamin D receptor (VDR) and the Bcl2-Interacting Mediator of Cell Death (Bim), and that HL60 and U937 cell lines can model the underlying events.

## RESULTS

### Cytotoxicity of AraC to AML blasts, but not to normal mononuclear bone marrow (NBM) cells, is selectively increased by the combination of D2 and CA

**Figure 1:**
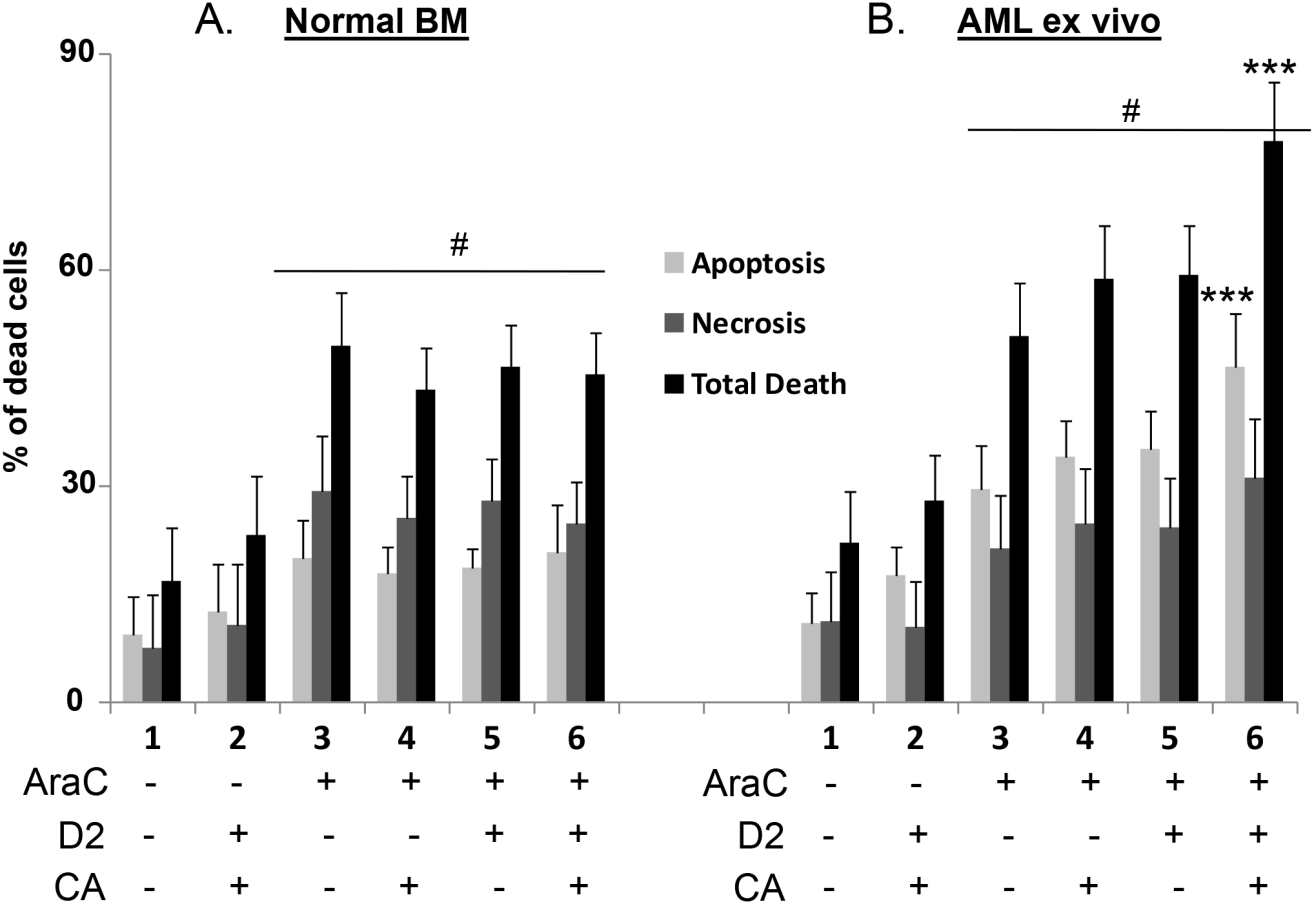
Comparison of the effects of D2/CA on AraC-induced cell death in Normal BM and AML ex vivo cells **A** and **B.** The treatment groups were: 1. Vehicle control for 96 h. 2. D2+CA- 96 h. 3. AraC-72 h-then cells washed and placed in normal medium for −96 h. 4. AraC-72 h-then cells washed and placed in CA for 96 h. 5. AraC-72 h-then cells washed and placed in D2 for 96 h. 6. AraC-72 h-then cells washed and placed in D2+CA for 96 h. The concentrations of treatment agents and other experimental details are described in in Materials and Methods. #= p<0.05 when compared with untreated control. ***=p < 0.001, compared with AraC-only treated group, n=5 for normal BM cells, n=9 for AML ex vivo cells. See also Supplemental Tables S1, S2 and S3.

While the responses of cells in established culture to the enhancement of AraC cytotoxicity by differentiation agents can suggest a therapeutic effect, this needs to be validated by conditions that more closely approximate the projected application in the clinic. Thus, we studied the responses to this treatment regimen (AraC-D2/CA) of AML blasts obtained from patients and placed in primary culture. Samples from nine consecutive patients provided blasts for the determination of cell viability by Annexin V staining (Figure 1 and Table 1), and six of these had also sufficient material for Trypan blue (TB) exclusion to be measured (Table S1). Samples of normal bone marrow (NBM) were also obtained from consented research subjects with non-neoplastic diseases, or healthy volunteer donors, for comparison with the effects of AraC-D2/CA treatment and showed no enhancement of AraC-induced cytotoxicity by TB exclusion (Table S1).

**Table 1:** Clinical and enhancement of apoptosis data for the AML ex vivo specimens

Patient	Classifcation	Age	Sex	Racial/Ethnic	% Enhancement	Diagnosis	Comments
**1**	**AML-M2**	84	M	Caucasian	99%	AML with partial myeloid maturation	Molecular markers not performed
**2**	**AML-M2**	51	M	Hispanic	67%	AML with partial differentiation	Wild Type FLT-3; Wild Type C/EBP alpha
**3**	**AML-M1**	63	M	Caucasian	90%	AML with minimal differentiation	No molecular abnormalities found
**4**	**AML-M4**	31	M	African-American	48%	Acute Myeloid Leukemia – FAB M4 with eosinophilia	Karyotype 46, XY, inv(16)
**5**	**AML-M2**	50	M	Hispanic	51%	Myeloid Leukemia with multilineage dysplasia	Presence of bcr-abl; Ph+ positive AML
**6**	**AML-M2**	79	M	Caucasian	45%	Acute Biphenotypic AML with lymphoid and myeloid markers	CD4 partial expression
**7**	**AML-M4**	60	M	Caucasian	39%	AML, myelomonocytic, without inversion of chromosome 16	No karyotypic abnormalities found by FISH
**8**	**AML-M4**	82	F	Caucasian	86%	AML with multilineage dysplasia	CD11b, CD14, CD33, HLA-DR, CD13, CD56, CD15 positive
**9**	**AML-M5**	46	M	Caucasian	124%	AML with an underlying myelodysplasia	CD34, CD117, CD68 positive

% Enhancement = (a-b)/bx100; a = Apoptosis when blasts exposed to AraC-D2/CA;

b = Apoptosis when blasts exposed to AraC alone. Mean enhancement of apoptosis was 72%.

As shown in Figure 1, panel A, cultures of NBM cells treated for 3 days with AraC at 100 nM, a clinically achievable concentration [20], had a significant proportion of cells which underwent cell death characterized by a lack of staining by Annexin V but were permeable to Propidium Iodide (PI), here referred to as “necrosis”. Apoptosis, as determined by Annexin V positivity was also observed. However, in NBM cells apoptosis, necrosis or total cell death (TD) were not increased by a subsequent exposure to the D2/CA combination (compare Figure 1A, lane 3 with lane 6).

In contrast, while AraC induced a similar extent of cell death in AML blasts as in NBM cells subjected to the same regimen, when D2/CA combination was added to AML cells, apoptosis, “necrosis”, and their sum (total death, TD) further increased by 72%, 29% and 44%, respectively (Figure 1B, and Table 1). These increases were statistically significant in AML cells for apoptosis and for total cell death, but not for necrosis (Figure 1B), which as detected here may represent a conglomerate of several other forms of cell death [21]. It is clear that AraC is toxic to both normal and neoplastic cells, but the D2/CA enhancement of AraC toxicity is apparent only in the neoplastic cells (Figure 1A and 1B; Table S1). In these cells the enhancement of death by apoptosis was highly significant (p< 0.001), but the increase in death by necrosis was not significant (Figure 1B).

Interestingly, there was no predilection of D2/CA enhancement for FAB subtypes. The responses of the four patients studied here with FAB-M2 ranged from 45% to 99% enhancement of AraC cytotoxicity, and in the FAB subtypes with partial differentiation (M4 and M5) the responses ranged was 39%-124% enhancement (Table 1). Of note, samples from every patient tested showed at least some response to D2/CA over that of AraC alone (Table 1).

We also tested if either D2, or CA alone is sufficient to enhance AraC cytotoxicity. The addition of either compound alone increased the cytotoxicity of AraC to AML blasts significantly but modestly (Tables S1 and S2). However, the enhancement by the D2/CA combination was quantitatively much greater, and statistically more significant. In contrast, NBM samples did not show significant changes following similar treatments (Table S2).

Thus, the data strongly support the selectivity of the enhancement regimen to malignant cells, and the necessity to use a combination of two additional differentiating agents for an optimal increase of the cytotoxicity of AraC to AML blasts.

### In AML blasts the AraC-D2/CA-induced cell death is associated with increased DNA damage and higher levels of DNA Damage Response (DDR) markers

**Figure 2:**
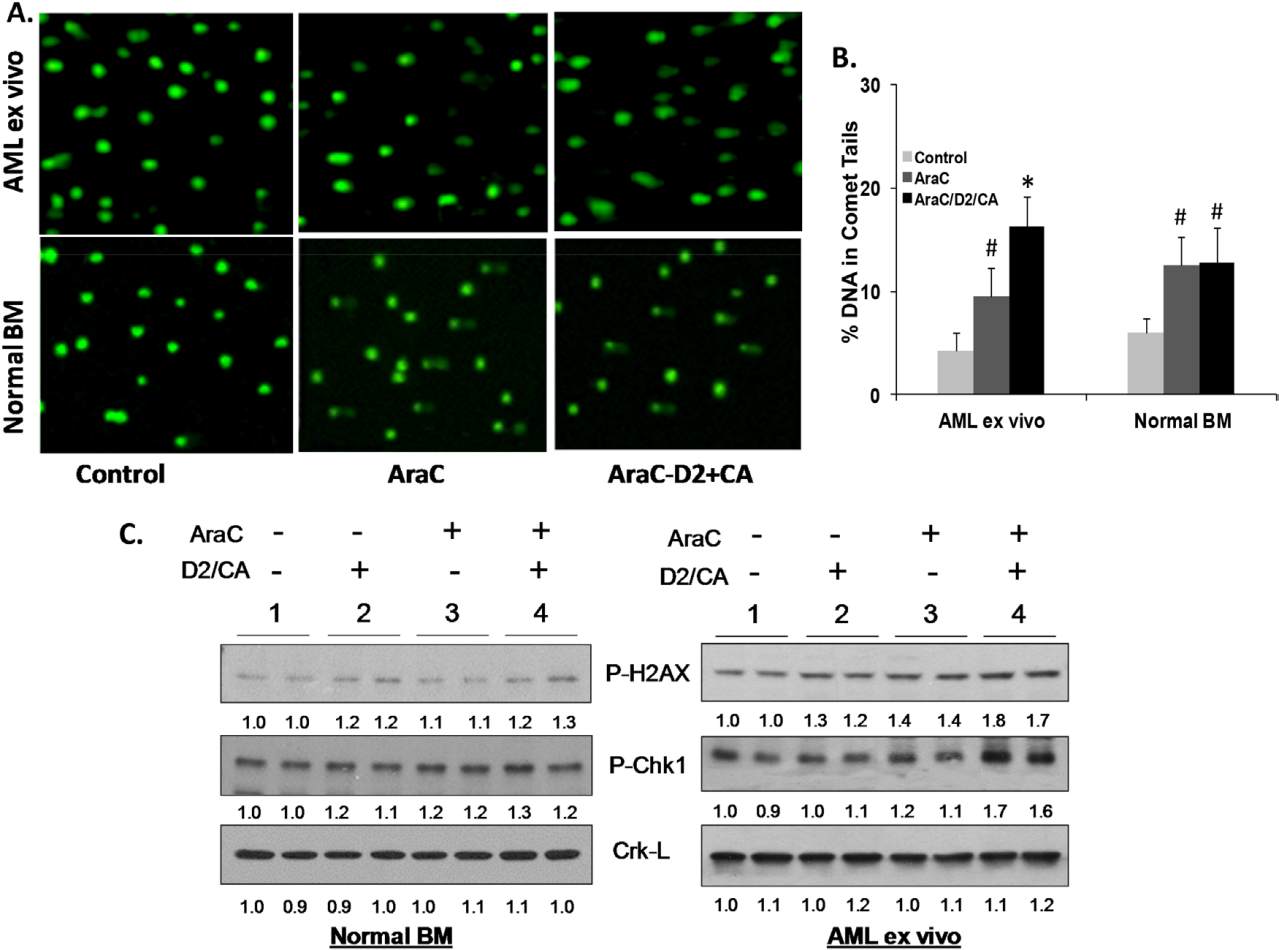
Enhancement of cell kill correlates with the evidence of DNA damage and with DNA Damage Response **A.** Comet assays of DNA damage. Representative images of the tailing of damaged DNA show that AML cells ex vivo (Top row) show DNA damage when exposed to AraC (100 nM for 72 h alone and maintained in normal medium for additional 96 h, center panels). DNA damage is increased when followed by D2 (100 nM) together with CA (10 μM) for 96 h (right side panels). In normal bone marrow (NBM) cells these changes were minimal (Bottom row). **B.** Quantitation of comet tails, as described in M & M. # = p <0.05 when compared to untreated control; * = p < 0.01, compared with AraC treated group; n=3 patients. **C.** Western blots showing protein levels of molecular markers of DNA damage (P-H2AX) and of DNA damage signaling (P-Chk1). Duplicates of each experimental group were run, as shown by the horizontal line above the signals. Note that AraC alone has only a modest effect on these signals, but the addition of D2/CA to AML blasts markedly increases the AraC effect. In contrast, there is little effect of these treatments on normal BM cells, and the D2/CA combination has no detectable effect on vehicle–treated control cells. Crk-L signal provided a loading control for the Westerns, which was used to determine the corrected OD for each signal, listed below the blot.

We documented the D2/CA-increased DNA damage in AML, but not in NBM cells by single-cell electrophoresis known as the comet assay. Under alkaline conditions this method detects a variety of genotoxic changes, including double and single DNA strand breaks and incomplete excision repair sites [22]. Figure 2A illustrates the increased number of comet tails when D2/CA follow AraC exposure of AML blasts, and the intensity of the comet tails was quantitated and is shown in Figure 2B. While no significant changes were seen in AraC-D2/CA treated NBM, AML blasts showed enhancement of the AraC-induced DNA damage by D2/CA.

DNA damage was also demonstrated on the molecular level by components of the DDR cascades. We chose two of these, P-H2AX and P-Chk1 [23, 24], to further document the enhancement phenomenon (Figure 2C). NBM cells showed no significant increase in the expression of these markers of DDR by the addition of D2/CA following AraC. In contrast, in AML blasts, the AraC alone-elicited modest increases in P-H2AX and P-ChK1 protein levels were enhanced by the addition of D2/CA (Figure 2C). Thus, these differentiation-inducers markedly increased AraC-induced DNA damage and DDR in AML blasts.

### Stimulation of monocytic differentiation in cells with AraC-induced DNA damage by the D2/CA combination

**Figure 3:**
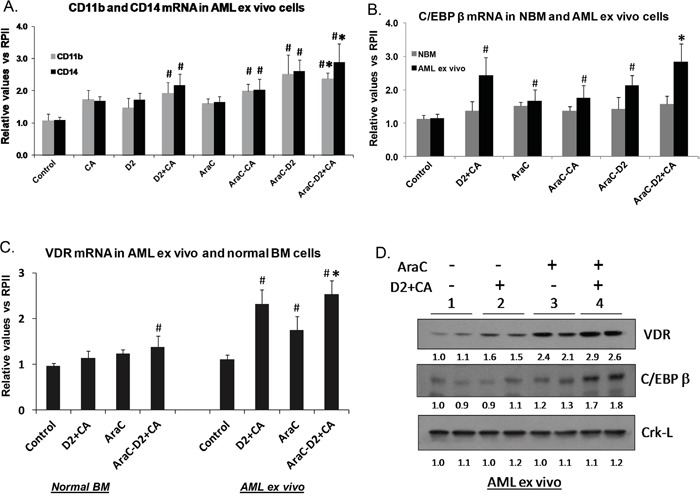
AML cells can initiate differentiation under cytotoxic conditions **A.** Effects of D2 and CA, alone or in combination, on the expression of the mRNA for monocytic differentiation markers CD11b and CD14, in untreated or AraC-treated cells. # = p < 0.05, when compared with control group.* = p < 0.05, when compared with AraC alone - treated group, n=3. **B.** qRT-PCR for C/EBP beta **C.** VDR mRNA levels were enhanced by AraC-D2/CA combination in AML blasts when compared to AraC-alone, but not in normal bone marrow cells. Symbol # = p, 0.05 when compared to control group. **D.** Western blots showing the upregulation of two molecular markers of monocytic differentiation (VDR and C/EBPbeta) following the exposure to D2/CA combination, AraC alone, and those added sequentially. Note the enhancement of monocytic differentiation markers when 1-D2/CA combination follows AraC treatment. The Crk-L signal provides a loading control for the Westerns, which was used to determine the corrected OD for each signal, listed below the blot.

AML blasts and cell lines derived from them can be induced to differentiate by several compounds including VDDs, alone or in combination [11, 16, 25–27]. However, cell differentiation may be masked by cytotoxicity, particularly for the determination of cell surface protein markers such as CD11b and CD14. Accordingly, to demonstrate the activation of the differentiation program in AraC-treated cells, we determined the mRNA levels of these markers by qRT-PCR, and found evidence of at least the initial stages of differentiation and its signaling. Increased mRNA levels of both CD11b and CD14 markers in AML blasts, though modest, suggested monocytic type of differentiation (Figure 3A). This was supported by the increased expression of the C/EBPβ transcription factor (TF), linked to signaling of monocytic differentiation [28] in AML blasts, but not in non-differentiating normal bone marrow cells treated in the same way (Figure 3B). Similarly, the expression of the vitamin D-regulated TF VDR was found to be enhanced at mRNA and protein levels by the AraC-D2/CA combination (Figure 3C and 3D). Thus, the enhanced DNA damage to AraC-treated AML blasts correlates with the increased expression of monocytic differentiation markers.

**Figure 4:**
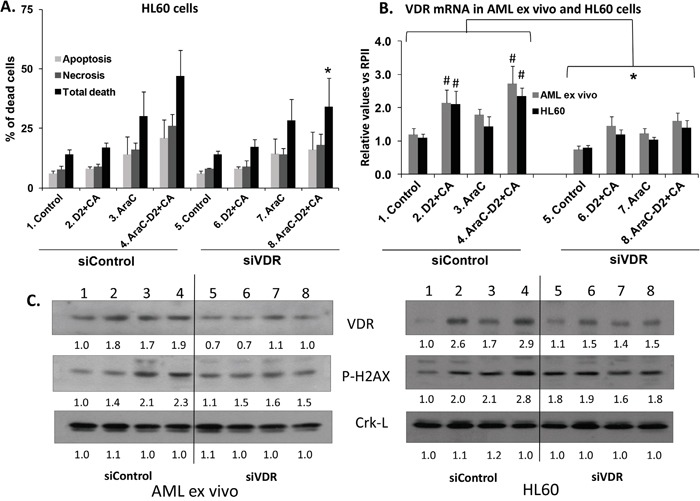
Knock down of VDR reduces the enhancement of cell death and DDR induced by D2/CA in AraC-treated AML ex vivo blasts and HL60 cells **A.** Near abrogation by siVDR of the enhancing effect of D2/CA on AraC-induced cell death. Each sample was divided and exposed to either Control siConstruct, or siVDR, as described in Materials and Methods. **B.** Levels of VDR mRNA following treatment with a siVDR pool. **C.** Westerns showing signal diminution following VDR knock down in both AML ex vivo blasts and HL60 cells. * = p< 0.05 for all values under the bracket when compared to the corresponding siControl; # = p< 0.05 compared to the untreated control. n=3.

To determine if VDR signaling is essential for the optimal effect of the AraC-D2/CA regimen on AML cell death, we knocked down the cellular levels of VDR. Using siVDR we demonstrated in ex vivo blasts and HL60 cells a significant, though partial (~ 40%), decrease of VDR levels at both mRNA and protein levels (Figure 4B and 4C). This effect correlated with siVDR-induced reduction in DDR, as measured by P-H2AX protein levels (Figure 4C). Measured by TB exclusion, AraC only-induced cell death was not affected by VDR knock down, but was markedly reduced (compared to siControl transfected cells) when D2/CA was added to AraC-treated AML cells (Supplementary Table S3A and S3B). Since a limited number of ex vivo blasts were available for this experiment we used only TB determinations on ex vivo cells, but similar effects on total cell death were obtained by Annexin V staining of HL60 cells, including the statistical significance of the inhibition of the D2/CA effect by siVDR (Figure 4A). Thus, VDR signaling is important for the optimal effect of the AraC-D2/CA regimen on cell death, though other factors may also be necessary.

### Cell death by apoptosis induced by AraC-D2/CA is caspase dependent

To better document that apoptosis is the central cell death process in the enhancement phenomenon, we studied the effect of several inhibitors of caspase activity. The results show that the enhancement of apoptosis and therefore total cell death in ex vivo AML blasts was abrogated by the specific caspase 3 inhibitor DEVD-CHO [29], but the caspase 1 inhibitor Z-WEHD-fmk [30] had only a minor effect (Table 2A, marked decreases are bolded). No significant changes were noted in NBM cells. Since only one normal and one malignant ex vivo specimen were available for this experiment, we used cell lines HL60 and U937 to investigate if AML cell lines show a similar effect as ex vivo samples, and if so, to obtain the statistical significance (Table 2B, bolded numbers designate p < 0.05). These experiments confirmed that the enhancement by D2/CA of AraC-induced cell death is principally caspase 3-dependent, consistent with its role as an executioner of apoptosis (Table 2B). Thus, we demonstrate that caspase-dependent apoptosis is an integral part of cell toxicity studied here.

Table 2Involvement of caspases in the enhancement of AraC cytotoxicity

**Table:** A

	AML ex vivo (%)			Normal BM (%)		
Treatment	Apoptosis	Necrosis	Total death	Apoptosis	Necrosis	Total death
Control-96 hr	1.0	1.0	2.0	8.4	4.6	13.0
AraC-72hr-medium-96 hr	29.7	11.0	40.7	40.2	23.6	63.8
AraC-72hr-D2+CA-96hr	51.5	11.3	62.8	43.6	21.8	65.4
AraC-72hr-D2+CA-Casp3 inhibitor	**27.1**	11.9	**39.0**	38.8	19.1	57.9
AraC-72hr-D2+CA-Casp1 Inhibitor	48.0	11.4	59.4	41.0	14.7	55.7
AraC-72hr-D2+CA-Casp Control	46.8	15.8	62.6	44.8	20.5	65.3

One blast sample was exposed to these agents.

**Table:** B

	HL60 (%)			U937 (%)		
Treatment	Apoptosis	Necrosis	Total death	Apoptosis	Necrosis	Total death
Control-96 hr	3.3 ± 1.5	2.5 ± 0.3	5.7 ± 1.3	4.7 ± 0.9	2.5 ± 0.6	7.2 ± 1.4
AraC-72hr-medium-96 hr	13.3 ± 1.4	5.5 ± 0.3	18.8 ± 1.7	23.2 ± 4.5	8.6 ± 2.2	31.8 ± 7.5
AraC-72hr-D2+CA-96hr	31.7 ± 6.1	16.5 ± 1.0	48.2 ± 7.1	36.9 ± 4.4	17.4 ± 5.5	54.3 ± 6.6
AraC-72hr-D2+CA-Casp3 Inhibitor	**18.8 ± 2.5**	11.4 ± 2.8	**30.2 ± 3.3**	**25.5 ± 8.6**	**8.7 ± 1.6**	**34.2 ± 9.6**
AraC-72hr-D2+CA-Casp1 Inhibitor	27.5 ± 7.5	13.5 ± 3.5	41.0 ± 4.0	31.6 ± 4.7	14.7 ± 1.7	46.3 ± 4.7
AraC-72hr-D2+CA-Casp Control	30.1 ± 3.3	16.4 ± 1.6	46.5 ± 3.9	36.1 ± 6.5	19.1 ± 5.2	55.2 ± 7.8

The experiments were repeated at least three times.

### Apoptotic cell death induced by AraC-D2/CA is associated with enhanced upregulation of the pro-apoptotic Bim

In view of Annexin V apoptosis data (Figure 1) and the above demonstration of the caspase-dependent apoptotic process (Table 2), we focused on the role of Bim, a BH3-only protein expressed most prominently by cells of hematopoietic origin [31]. Bim has emerged as a key pro-apoptotic protein in the initiation of the intrinsic apoptotic pathway under many conditions, including treatment of AML cells with therapeutic agents (eg [32–37]).

**Figure 5:**
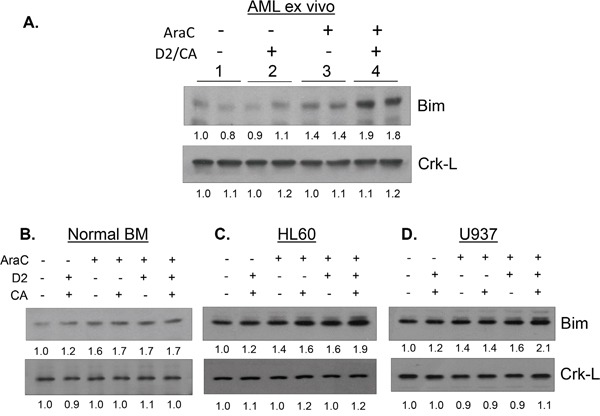
Effect of D2/CA, AraC and their combination on the expression of the pro-apoptotic protein Bim **A.** AML ex vivo blast cells, **B.** Normal BM cells, **C.** HL60 cells and **D.** U937 cells. Crk-L protein was used as internal control for loading.

When Bim expression was examined in this study we found a marked, statistically significant, increase in Bim mRNA in AraC-treated cells, ex vivo and in AML cell lines, which was further increased by D2/CA (Table 3). However, while AraC alone also significantly increased Bim expression in NBM cells, the addition of 1-D2/CA did not significantly increase it further, consistent with no increase cell death of normal cells (Figure 1A and Table 2A). Western blots confirmed the upregulation of Bim at protein level in AraC-only treated cells in parallel with the RT-PCR data (Figure 5 and Table 3). However, although D2/CA also increased Bim mRNA expression in all AML cell types studied, this was not reflected at the protein level in the D2/CA-only groups (Table 3 and Figure 5), even though when administered after AraC, both mRNA and protein levels were increased compared to AraC alone (p <0.02, Table 3). This suggests that Bim is upregulated in this system principally at the transcriptional level, but in AML cells that do not manifest toxic effects, the translation to protein is impaired, or the degradation of Bim mRNA is rapid.

**Table 3:** Levels of Bim mRNA in AML and Normal BM cells following treatment with D2/CA, and AraC alone or followed by differentiation agents

	AML ex vivo			Normal BM		
	Mean ± SD	vs Control	vs AraC	Mean ± SD	vs Control	vs AraC
Treatment	Bim	P value	P value	Bim	p value	p value
Control-96 hr	1.19 ± 0.17			1.13±0.14		
D2+CA-96 hr	1.65 ± 0.17	**0.003**		1.61±0.11	**0.004**	
AraC-72hr-medium-96hr	2.15 ± 0.35	0.004		1.83±0.16	0.039	
AraC-72hr-CA-96hr	1.86 ± 0.13	0.001	0.100	1.77±0.17	0.003	0.689
AraC-72hr-D2-96hr	1.79 ± 0.17	0.005	**0.013**	1.81±0.17	0.054	0.926
AraC-72hr-D2+CA-96hr	2.93 ± 0.43	0.001	**0.013**	1.85±0.24	0.080	0.901

	HL60			U937		
	Mean ± SD	vs Control	vs AraC	Mean ± SD	vs Control	vs AraC
Treatment	Bim	p value	p value	Bim	p value	p value
Control-96 hr	1.21 ± 0.20			1.24 ± 0.22		
D2+CA-96 hr	2.07 ± 0.12	0.015		1.70 ± 0.14	0.047	
AraC-72hr-medium-96hr	2.34 ± 0.29	0.002		2.01 ± 0.21	0.045	
AraC-72hr-CA-96hr	2.04 ± 0.15	0.046	0.322	1.92 ± 0.23	0.065	0.774
AraC-72hr-D2-96hr	2.2 ± 0.14	0.033	0.638	1.84 ± 0.17	0.115	0.368
AraC-72hr-D2+CA-96hr	2.72 ± 0.22	0.003	**0.013**	2.52 ± 0.25	0.041	**0.021**

The values shown represent the ratio of Bim vs RPII as described in Materials and Methods. There was one sample of ex vivo specimens each, and HL60 and U937 were performed in three separate experiments.

**Figure 6:**
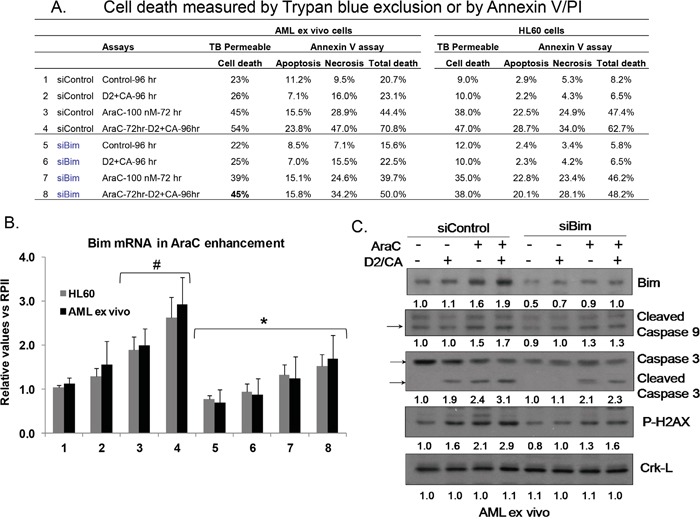
Effects of Knock down of Bim on cell death enhancement by D2/CA, caspases, and a DDR marker **A**. Cell death following knock down of Bim as measured by Trypan blue exclusion or by Annexin V/PI assay. **B.** Levels of Bim mRNA following the treatment with a siBim pool oligonucleotides. #=p< 0.05 compared to untreated controls; *= p< 0.05 compared to the corresponding siControl **C.** Western blots for Bim protein, activated caspase 9, caspase 3, and P-H2AX, and the loading control, Crk-L.

In order to establish that Bim upregulation was functionally related to the execution of apoptosis, we transfected a pool of Bim siRNA and control siRNA into AML blasts ex vivo and into HL60 cells, and subjected the transfected cells to the AraC-D2/CA procedure. This reduced the D2/CA effect of enhancing AraC toxicity in AML blasts, as measured by Trypan blue exclusion (from 54% to 45%), or by Annexin V/PI (from 71% to 50%) (Figure 6A). Similar reductions were observed in HL60 cells. Bim siRNA effectively reduced Bim mRNA level (Figure 6B), and this was also seen at protein level (Figure 6C, top row). Further, siBim reduced the levels of activated Caspase 9 and caspase 3, demonstrating that Bim regulates this caspase cascade in AML blasts (Figure 6C). Also, while the level of P-H2AX, the marker of DDR, was upregulated by the AraC-D2/CA exposure, this was reduced by siBim, implicating Bim directly in DNA damage (Figure 6B).

## DISCUSSION

In this report we have elucidated several important details of the underlying mechanism of cell death enhancement by D2/CA in blasts with DNA damage induced by AraC. Principally, we show that the vitamin D-regulated transcription factor VDR is an essential component of cell death enhancement following treatment with AraC, as VDR is not only expressed at higher levels when D2/CA exposure follows AraC, but experimental reduction of VDR levels reduces D2/CA-induced cell death. This suggests that the differentiation programs initiated by VDR interfere with DNA damage repair in AraC-treated AML cells. Further details of this mechanism will require extensive additional studies, as DNA repair depends on numerous components, and what tips DNA repair over to cell death is not precisely clear [43–45]. Importantly, in this study we demonstrate that AML blasts, when obtained directly from patients with this disease, show the same vulnerability to vitamin D-based modification of the standard therapy as cells from the established AML cell lines. This can provide a basis for developing this regimen in further studies as of the modification of AraC therapy in the clinic. The marked increases in “comet tails” shown in Figure 2A illustrate the extensive blast cell death due to AraC-D2/CA. While in the current in vitro short-term exposure the average increase in apoptotic cell death due to D2/CA was 72%, with the majority more than 50% higher than AraC alone, in a clinical setting the standard treatment with AraC, if followed by several weeks of D2/CA, can be expected to increase blast cell death even more robustly. As noted above, although the normal bone marrow is affected by AraC alone, it will not be further affected by D2/CA addition, while every AML specimen studied showed some D2/CA-induced enhancement.

We have also elucidated additional key details of the mechanisms that enhance cell death. Principally, we show that the caspase cascade is an integral part of the cell death enhancement. In ex vivo blasts caspase 3 inhibitor completely abrogated the enhancement in the sample studied, while caspase 1 inhibitor had very little effect. In AML cell lines caspase 3 inhibitor also almost completely inhibited the cell death enhancement, but inhibition of caspase 1 had no discernible effect (p > 0.05). In agreement with these results regarding cell death, lowering the levels of activated caspase 9 and 3 by siBim reduced the DDR, as shown by the lower level of P-H2AX (Figure 6C).

The discrepancy between the increased mRNA but no increase in protein levels of Bim in D2/CA-only treated AML cells can be explained by the previous observation that Bim mRNA is known to be targeted by miR32, which prevents its translation [38]. It should also be noted that although the Bim gene can be alternatively spliced into various isoforms, there are only three major transcription variants with different sized proteins [39], and the form enhanced by D2/CA is predominantly “extra-large”, ie Bim_EL_ (see Figure 5A). While the data obtained here suggest that Bim_EL_ has an important role in the activation of caspases that enhance AraC-induced death of AML cells exposed to D2/CA, it is likely that several other regulators of apoptosis also participate in these events. There is increasing realization that although VDDs on long term administration may be efficient cancer chemopreventative agents [40], but the use of VDD in cancer therapy will need combination with other agents. For instance, a recent report of survival improvement in patients with AML or Myelodysplastic Syndrome, who were in remission achieved with standard chemotherapy, and who received maintenance treatment with agents that included differentiating agents [41], is consistent with the hypothesis that the exposure to a VDD after cellular DNA damage is necessary for improved outcome of therapy. Also, recent data from clinical observations showing that patients with newly diagnosed AML who had low (<32 ng/mL) serum vitamin D3 levels have poorer survival than those with normal vitamin D3 levels [42]. Additionally, there are numerous recent reports that in various in vitro or in vivo models of solid tumors vitamin D3 or other VDDs enhance the effectiveness of cytotoxic drugs (eg [43–47]). However, our approach differs from those, as a non-toxic antioxidant [19], carnosic acid, is added to potentiate the transcriptional activity of the VDD, doxercalciferol, and this is selective for malignant AML blasts.

It is also of interest that while the usual effect of VDDs on solid tumors is induction or enhancement of apoptosis (eg, [48, 49]), in myeloid hematopoietic [50] and skin derived cell cultures, eg keratinocytes [51] VDDs have a protective effect, that has been attributed to activation of Akt [52]. This raises the distinct possibility that the extension of the mechanistic studies described here may help to solve the puzzle how DNA repair fails, by comparing the effects of VDDs on undamaged AML cells with their effects on cells with already damaged DNA.

Collectively, the data obtained on the enhancement of AraC-induced cell death by D2/CA already suggest that these preclinical results may be translated into an advance in the existing, but usually only transiently effective, chemotherapy for AML.

## MATERIALS AND METHODS

### Chemicals and antibodies

Arabinocytosine and Doxercalciferol (1α-hydroxyvitamin D_2_; 1-D2) were purchased from Sigma-Aldrich (St. Louis, MO). Carnosic acid (CA) was purchased from Enzo Life Sciences, Inc. (Farmingdale, NY). The antibodies used for Western blots were: Bim (sc-8625), VDR (sc-1008), C/EBP beta (sc-150) and Crk-L (sc-319) were obtained from Santa Cruz Biotechnology (Dallas, TX). Phospho-Ser139-H2AX (#9718), phospho-Ser345-Chk1 (#2348), caspase 3, caspase 9 and HRP-linked anti-rabbit (#7074) antibodies were from Cell Signaling Technologies (Danvers, MA). The siRNA transfection reagents were from Santa Cruz Biotechnology (Dallas, TX), and Caspase 3 inhibitor (Z-DEVD-FMK) from Santa Cruz Biotechnology. Specific caspase 1 inhibitor (Z-WEHD-FMK) and negative control for caspase inhibitors (Z-FA-FMK) were from Abcam (Cambridge, MA). AML cells were pretreated with 100 nM AraC for 72 h, then incubated with 10 μM of caspase inhibitors for 1 h before adding other agents.

### Isolation of mononuclear cells from peripheral blood or bone marrow samples and culture

Specimens of peripheral blood and bone marrow were obtained according to IRB protocol during diagnostic procedures from 9 patient volunteers with different FAB subtypes of AML (Table 1). Additional five volunteers provided normal bone marrow samples. Mononuclear cells were isolated from the specimens by using Histopaque-1077 (Sigma-Aldrich) gradient centrifugation, as previously described [26]. Isolated mononuclear cells were divided into a group exposed for 72 h to 100 nM AraC or the vehicle (0.1% DMSO). The cells were washed with control medium after this 72 h pretreatment, and each of these groups was further divided to add D2 (100 nM), or CA (10 μM), or both, for 96 h. Cell viability was determined by Trypan blue (TB) exclusion using a Neubauer hemocytometer as described before [53].

### Cell lines

Two AML cell lines, HL60 (cultured from a patient with acute promyeloblastic leukemia [54], and U937 (monocytes from histiocytic lymphoma) [55], were cultured as described [53].

### Flow cytometry analysis for Annexin V and propidium iodide staining

Experimental cells were washed twice with 1xPBS, then resuspended in the binding buffer, containing 0.14 M NaCl and 2.5 mM CaCl_2_, pH 7.5, and incubated with 50 μg/ml Annexin V-FITC (kit from Sigma) and 20 μg/ml propidium iodide in 1x binding buffer at room temperature in the dark for 15 minutes, and immediately analyzed by flow cytometry (EPICS XL). Annexin V-positive/PI-negative cells were considered as early apoptotic, cells both Annexin V and PI positive, as late apoptotic, and Annexin negative but PI positive as “necrotic”, likely a variety of caspase independent modes of cell death [21].

### Comet assay analysis

DNA damage within cells was measured by the comet assay [56]. Briefly, mononuclear cells isolated from AML patients samples, or normal BM samples, were treated with AraC or AraC/1-D2/CA for indicated times. This assay was performed according to the manufacturer's recommended procedure (Cell BioLabs, San Diego, CA). Briefly, AML cells were washed twice with 1xPBS, then 10,000 cells were mixed with low melting-point agarose gel and pipetted on the “Comet Slide”. The cells transferred to the slide were maintained for 15 minutes at 4°C in the dark. The slide was then immersed in lysis buffer for 30 minutes at 4°C in the dark, to relax and denature the nuclear DNA. Next, the slide was transferred into a horizontal electrophoresis tank in TBE buffer, and ran for 15 minutes at 30 volts, then rinsed three times with distilled water and once with 70% ethanol. Finally, the dried slide was stained with Vista Green DNA Dye for 15 minutes at room temperature. Cells on the slide were visualized and photographed using a fluorescent microscope. The images of cells were analyzed with the Opencomet Software, a plug-in for the image processing platform, ImageJ [57]. The percentage of DNA in comet tails was selected as a measure of DNA damage. In each slide, obtained from at least three independent experiments, 50 stained cells were measured, scored and analyzed.

### Quantitative real time PCR analysis

Total RNA was extracted by using Trizol (Invitrogen, CA) according to manufacturer's protocol as described before [53]. Quantitative RT-PCR was carried out using a ABI SYBR Green master kit (Applied Biosystems, Foster City, CA). Fold changes of mRNA levels in target gene relative to the RNA polymerase II (RPII) control were calculated by relative quantification analysis. Primers used for Bim were: upstream 5′-AGTTCTGAGTGTGACCGAGAAGGT-3′, downstream 5′-TCCTGTCTTGTGGCTCTG TCTGTA-3′; CD11b, upstream 5′-GCAAGTGTCTGTGTGCAAGTGTGT-3′, downstream 5′-TCAGTGGAGAGAAGCTGCTGTGTT-3′, CD14, upstream 5′-AAC TCCCTCA ATCTGTCGTTCGCT-3′, downstream 5′-GGGCAAAGG GTTGAATTGGTCGAA-3′, C/EBP beta, 5′-GTTCTTGACGTTCTTCGGCCG-3′ and 5′-TGGACAAGCACAGCGACGAGT-3′; For VDR: forward primer, 5′-CTTCAGGCGAAGCATGAAGC-3′; reverse primer, 5′-CCTTCATCATGCCGATGTCC-3′ for RP II, upstream 5′-GCACCA CGTCCAATGACAT-3′, downstream 5′-GTGCGGCTGCTTCCATAA-3′. The quality of PCR products were monitored using post-PCR melting curve analysis.

### Western blotting

Western blotting was performed using 50 μg of total cell extracts as described [19]. Each membrane was stripped and reprobed for Crk-L to serve as an internal control for loading. The optical density (OD) of each band was quantitated using ImageQuant 5.0 program (Molecular Dynamics, Sunnyvale, CA).

### Caspase inhibition

Cells were pretreated with 100 nM cytarabine for 72 h, then 10 μM of Caspase 3 inhibitor (Z-DEVD-FMK), caspase 1 inhibitor (Z-WEHD-FMK), or a negative control caspase inhibitor (Z-FA-FMK) were added 1 h before addition of D2 and carnosic acid for 96 h. Cells were harvested to check the effects of specific caspase inhibition on AraC/D2/CA-induced cell death by Annexin V/PI as described below.

### Knockdown of VDR or Bim expression

Mononuclear or HL60 cells were transfected with 10 μM of VDR siRNA or Bim siRNA or scrambled Control siRNA, using Endo-Porter delivery reagent from Gene Tools Inc (Philomath, OR) before exposure to other agents. The cells were allowed to recover in RPMI 1640 medium with 10% FCS for 24 h, and then were exposed to the indicated compounds for the indicated times. The reduction of target protein or mRNA by siVDR or siBim transfection in the enhancement group was approximately 40%, an efficiency consistent with other reports in the literature for AML cells which are difficult to transfect.

### Statistical analysis

Each experiment was repeated 3-6 times. The results are presented as the mean ± SD. Statistical significance of the differences between mean values was performed by a 2-tailed Student's T-test. All computations were done with an IBM-compatible personal computer using either Microsoft EXCEL program or GraphPad Prism 6 (LaJolla, CA).

## SUPPLEMENTARY TABLES








