# Supplementary material for: The role of VDR and BIM in potentiation of cytarabine–induced cell death in human AML blasts
Source: Oncotarget. 2016 Apr 26;7(24):36447–60. doi: 10.18632/oncotarget.8998 (PMC5095012; doi:10.18632/oncotarget.8998)
Supplement: Supplementary file 1 [file oncotarget-07-36447-s001.pdf]

The role of VDR and BIM in potentiation of cytarabine–induced cell death in human AML blasts

SUPPLEMENTARY TABLES

Supplementary Table S1: Total Cell Death (TD) as determined by Trypan blue exclusion

A. Normal Bone Marrow (BM): Total cell death by cell membrane permeability.

B. AML blasts ex vivo: Total cell death by cell membrane permeability.

See Supplementary File 1

Supplementary Table S2: Statistical significance of the enhancement of AraC-induced total cell death by CA, D2 or their combination. Cell death was assessed by Annexin V staining

See Supplementary File 2

Supplementary Table S3: The role of VDR in induction of Total Cell Death (TD) as determined by Trypan blue exclusion

A. HL60 cells (Six individual experiments)

B. AML blasts ex vivo (Three patient samples)

See Supplementary File 3
