# Supplementary material for: The role of VDR and BIM in potentiation of cytarabine–induced cell death in human AML blasts
Source: Oncotarget. 2016 Apr 26;7(24):36447–60. doi: 10.18632/oncotarget.8998 (PMC5095012; doi:10.18632/oncotarget.8998)
Supplement: Supplementary file 2 [file oncotarget-07-36447-s002.ppt]

## Slide 1
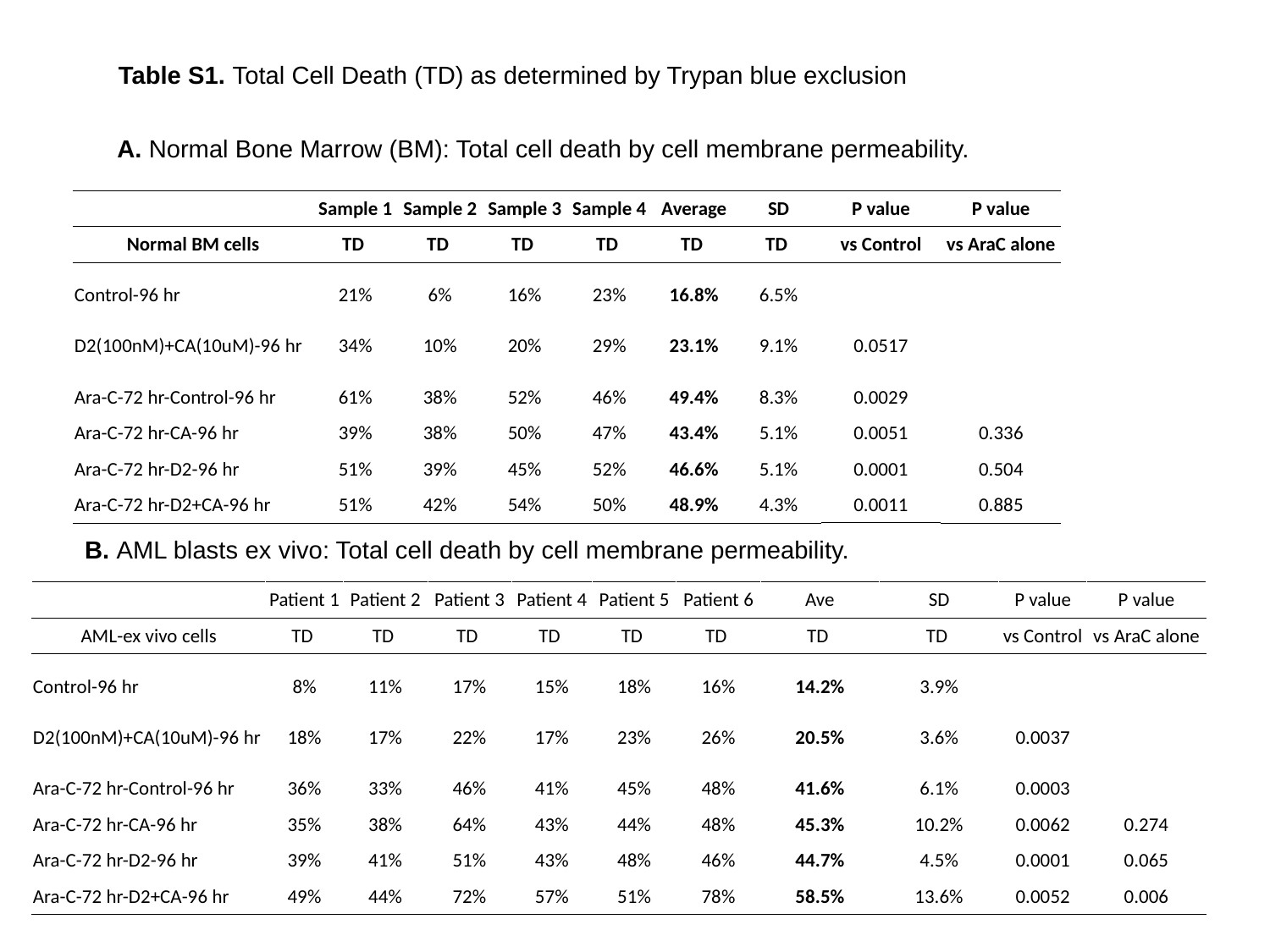

Table S1. Total Cell Death (TD) as determined by Trypan blue exclusion
A. Normal Bone Marrow (BM): Total cell death by cell membrane permeability.
| | Sample 1 | Sample 2 | Sample 3 | Sample 4 | Average | SD | P value | P value |
| --- | --- | --- | --- | --- | --- | --- | --- | --- |
| Normal BM cells | TD | TD | TD | TD | TD | TD | vs Control | vs AraC alone |
| Control-96 hr | 21% | 6% | 16% | 23% | 16.8% | 6.5% | | |
| D2(100nM)+CA(10uM)-96 hr | 34% | 10% | 20% | 29% | 23.1% | 9.1% | 0.0517 | |
| Ara-C-72 hr-Control-96 hr | 61% | 38% | 52% | 46% | 49.4% | 8.3% | 0.0029 | |
| Ara-C-72 hr-CA-96 hr | 39% | 38% | 50% | 47% | 43.4% | 5.1% | 0.0051 | 0.336 |
| Ara-C-72 hr-D2-96 hr | 51% | 39% | 45% | 52% | 46.6% | 5.1% | 0.0001 | 0.504 |
| Ara-C-72 hr-D2+CA-96 hr | 51% | 42% | 54% | 50% | 48.9% | 4.3% | 0.0011 | 0.885 |
B. AML blasts ex vivo: Total cell death by cell membrane permeability.
| | Patient 1 | Patient 2 | Patient 3 | Patient 4 | Patient 5 | Patient 6 | Ave | SD | P value | P value |
| --- | --- | --- | --- | --- | --- | --- | --- | --- | --- | --- |
| AML-ex vivo cells | TD | TD | TD | TD | TD | TD | TD | TD | vs Control | vs AraC alone |
| Control-96 hr | 8% | 11% | 17% | 15% | 18% | 16% | 14.2% | 3.9% | | |
| D2(100nM)+CA(10uM)-96 hr | 18% | 17% | 22% | 17% | 23% | 26% | 20.5% | 3.6% | 0.0037 | |
| Ara-C-72 hr-Control-96 hr | 36% | 33% | 46% | 41% | 45% | 48% | 41.6% | 6.1% | 0.0003 | |
| Ara-C-72 hr-CA-96 hr | 35% | 38% | 64% | 43% | 44% | 48% | 45.3% | 10.2% | 0.0062 | 0.274 |
| Ara-C-72 hr-D2-96 hr | 39% | 41% | 51% | 43% | 48% | 46% | 44.7% | 4.5% | 0.0001 | 0.065 |
| Ara-C-72 hr-D2+CA-96 hr | 49% | 44% | 72% | 57% | 51% | 78% | 58.5% | 13.6% | 0.0052 | 0.006 |
