# Supplementary material for: The role of VDR and BIM in potentiation of cytarabine–induced cell death in human AML blasts
Source: Oncotarget. 2016 Apr 26;7(24):36447–60. doi: 10.18632/oncotarget.8998 (PMC5095012; doi:10.18632/oncotarget.8998)
Supplement: Supplementary file 3 [file oncotarget-07-36447-s003.ppt]

## Slide 1
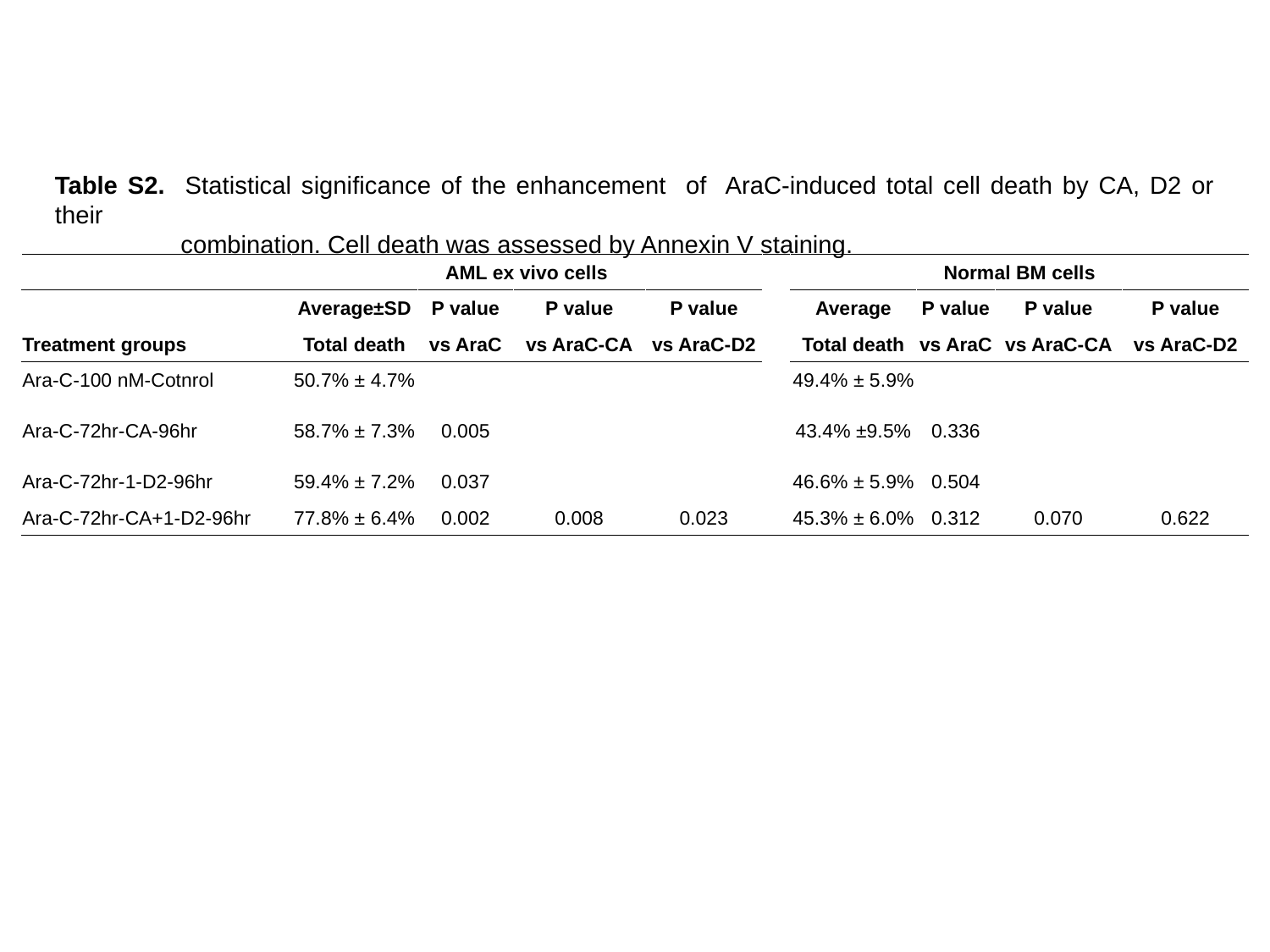

Table S2. Statistical significance of the enhancement of AraC-induced total cell death by CA, D2 or their
 combination. Cell death was assessed by Annexin V staining.
| | AML ex vivo cells | | | | | Normal BM cells | | | |
| --- | --- | --- | --- | --- | --- | --- | --- | --- | --- |
| | Average±SD | P value | P value | P value | | Average | P value | P value | P value |
| Treatment groups | Total death | vs AraC | vs AraC-CA | vs AraC-D2 | | Total death | vs AraC | vs AraC-CA | vs AraC-D2 |
| Ara-C-100 nM-Cotnrol | 50.7% ± 4.7% | | | | | 49.4% ± 5.9% | | | |
| Ara-C-72hr-CA-96hr | 58.7% ± 7.3% | 0.005 | | | | 43.4% ±9.5% | 0.336 | | |
| Ara-C-72hr-1-D2-96hr | 59.4% ± 7.2% | 0.037 | | | | 46.6% ± 5.9% | 0.504 | | |
| Ara-C-72hr-CA+1-D2-96hr | 77.8% ± 6.4% | 0.002 | 0.008 | 0.023 | | 45.3% ± 6.0% | 0.312 | 0.070 | 0.622 |
