# Supplementary material for: The role of VDR and BIM in potentiation of cytarabine–induced cell death in human AML blasts
Source: Oncotarget. 2016 Apr 26;7(24):36447–60. doi: 10.18632/oncotarget.8998 (PMC5095012; doi:10.18632/oncotarget.8998)
Supplement: Supplementary file 4 [file oncotarget-07-36447-s004.ppt]

## Slide 1
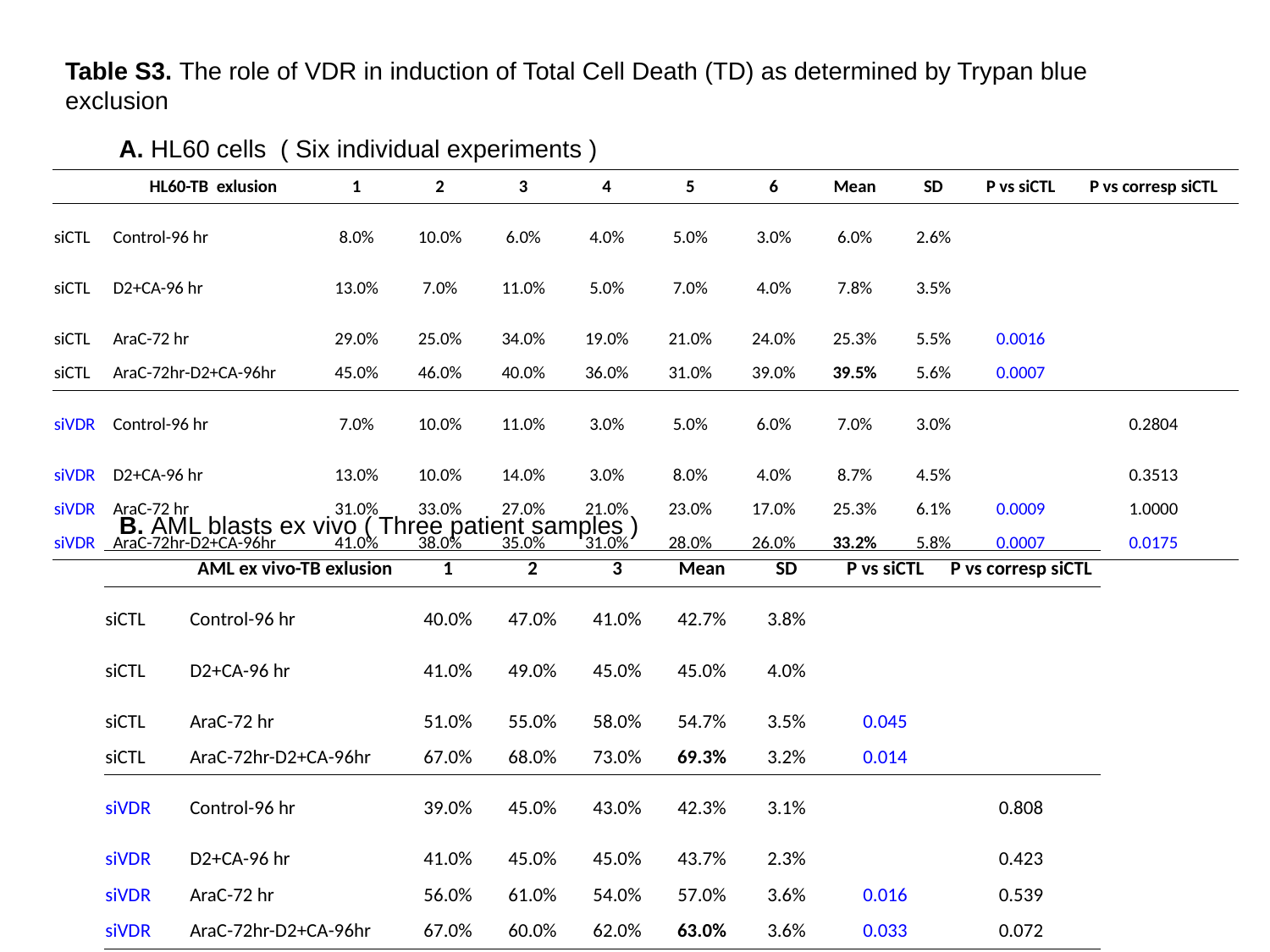

Table S3. The role of VDR in induction of Total Cell Death (TD) as determined by Trypan blue exclusion
A. HL60 cells ( Six individual experiments )
| | HL60-TB exlusion | 1 | 2 | 3 | 4 | 5 | 6 | Mean | SD | P vs siCTL | P vs corresp siCTL |
| --- | --- | --- | --- | --- | --- | --- | --- | --- | --- | --- | --- |
| siCTL | Control-96 hr | 8.0% | 10.0% | 6.0% | 4.0% | 5.0% | 3.0% | 6.0% | 2.6% | | |
| siCTL | D2+CA-96 hr | 13.0% | 7.0% | 11.0% | 5.0% | 7.0% | 4.0% | 7.8% | 3.5% | | |
| siCTL | AraC-72 hr | 29.0% | 25.0% | 34.0% | 19.0% | 21.0% | 24.0% | 25.3% | 5.5% | 0.0016 | |
| siCTL | AraC-72hr-D2+CA-96hr | 45.0% | 46.0% | 40.0% | 36.0% | 31.0% | 39.0% | 39.5% | 5.6% | 0.0007 | |
| siVDR | Control-96 hr | 7.0% | 10.0% | 11.0% | 3.0% | 5.0% | 6.0% | 7.0% | 3.0% | | 0.2804 |
| siVDR | D2+CA-96 hr | 13.0% | 10.0% | 14.0% | 3.0% | 8.0% | 4.0% | 8.7% | 4.5% | | 0.3513 |
| siVDR | AraC-72 hr | 31.0% | 33.0% | 27.0% | 21.0% | 23.0% | 17.0% | 25.3% | 6.1% | 0.0009 | 1.0000 |
| siVDR | AraC-72hr-D2+CA-96hr | 41.0% | 38.0% | 35.0% | 31.0% | 28.0% | 26.0% | 33.2% | 5.8% | 0.0007 | 0.0175 |
B. AML blasts ex vivo ( Three patient samples )
| | AML ex vivo-TB exlusion | 1 | 2 | 3 | Mean | SD | P vs siCTL | P vs corresp siCTL |
| --- | --- | --- | --- | --- | --- | --- | --- | --- |
| siCTL | Control-96 hr | 40.0% | 47.0% | 41.0% | 42.7% | 3.8% | | |
| siCTL | D2+CA-96 hr | 41.0% | 49.0% | 45.0% | 45.0% | 4.0% | | |
| siCTL | AraC-72 hr | 51.0% | 55.0% | 58.0% | 54.7% | 3.5% | 0.045 | |
| siCTL | AraC-72hr-D2+CA-96hr | 67.0% | 68.0% | 73.0% | 69.3% | 3.2% | 0.014 | |
| siVDR | Control-96 hr | 39.0% | 45.0% | 43.0% | 42.3% | 3.1% | | 0.808 |
| siVDR | D2+CA-96 hr | 41.0% | 45.0% | 45.0% | 43.7% | 2.3% | | 0.423 |
| siVDR | AraC-72 hr | 56.0% | 61.0% | 54.0% | 57.0% | 3.6% | 0.016 | 0.539 |
| siVDR | AraC-72hr-D2+CA-96hr | 67.0% | 60.0% | 62.0% | 63.0% | 3.6% | 0.033 | 0.072 |
